# Cultural Adaptation, Translation and Psychometric Validation of a Technology and eHealth Literacy Questionnaire Among Albanian Undergraduate Nursing Students

**DOI:** 10.3390/nursrep16040139

**Published:** 2026-04-15

**Authors:** Chrysi A. Fragkioudaki, Enkeleint A. Mechili, Petros Galanis, Evridiki Patelarou, Konstantinos Giakoumidakis, Athina E. Patelarou

**Affiliations:** 1 Department of Nursing, School of Health Sciences, Hellenic Mediterranean University, 71410 Heraklion, Greece; ddk107@edu.hmu.gr; 2 Department of Healthcare, Faculty of Health, University ‘Ismail Qemali’ of Vlora, 9401 Vlora, Albania; mechili@univlora.edu.al; 3 Clinical Epidemiology Laboratory, Faculty of Nursing, National and Kapodistrian University of Athens, 11527 Athens, Greece; pegalan@nurs.uoa.gr; 4 Laboratory of Evidence-Based Healthcare, Education and Clinical Protocols, Department of Nursing, School of Health Sciences, Hellenic Mediterranean University, 71410 Heraklion, Greecekongiakoumidakis@hmu.gr (K.G.)

**Keywords:** electronic health, nursing, validation, students, technology

## Abstract

**Background**: Rapid technological progress has transformed healthcare systems through integrating electronic health (eHealth) into clinical practice. Consequently, nursing students, upcoming healthcare professionals, face new challenges arising from this digital transition. Adequate technological skills and eHealth literacy are essential to meet the requirements of contemporary healthcare environments. The aim of this study was to translate, culturally adapt, and validate a questionnaire measuring technophilia, Internet use, eHealth literacy, and nursing students’ attitudes toward technology and eHealth. **Methods**: A cross-sectional validation study was conducted using a convenience sample of Albanian undergraduate nursing students. After forward and backward translation, the instrument’s construct validity was examined using exploratory factor analysis (EFA). Cronbach’s alpha assessed internal consistency, and the relationships among the four constructs were explored using structural equation modeling (SEM). **Results**: A total of 357 undergraduate nursing students participated in the survey. EFA identified a clear four-factor structure corresponding to Technophilia, Internet Use, eHealth Literacy, and Technology and Electronic Health in Nursing Education, with all items demonstrating satisfactory factor loadings. Internal consistency of the four scales ranged from 0.692 to 0.852, indicating generally satisfactory reliability. Although the SEM model fit was below the recommended thresholds, the results provide some evidence for relationships among the constructs. **Conclusions**: The findings provide preliminary evidence for the reliability and validity of the adapted instrument and set a baseline for assessing Albanian nursing students’ knowledge, skills, and attitudes regarding technology and eHealth literacy. Several strategies can be developed based on this evidence to prepare nursing students for technologically advanced healthcare systems.

## 1. Introduction

Over the past decades, healthcare systems have increasingly integrated digital technologies and electronic health (eHealth) into clinical practice to address both communicable and non-communicable diseases effectively [1,2]. Notably, there is a pressing need for healthcare professionals, including registered nurses, to have advanced digital skills and to become eHealth literate [3,4]. Traditional face-to-face care is increasingly supplemented or replaced by remote care using information and communication technologies (ICT) [4,5]. This change is motivated by the need to access quality and accurate health information or distant medical advice whenever necessary, especially during global health crises like the COVID-19 pandemic [2,6,7,8].

The ability to seek, access, and appraise health information from electronic resources for health-related problems is crucial for individuals and nursing students who will become future healthcare professionals, and they are among the largest groups in healthcare [3,4]. The significance of eHealth literacy is evident not only in the numerous research studies on the field worldwide, but also in its paramount role in effective healthcare delivery [9].

In line with global trends, Albania has laid the foundations for the digital transformation of its services. The country now provides Internet access to up to 80% of its citizens and mobile broadband to 85% of users. In comparison, the European averages are 89% and 87%, respectively [10]. These interventions are supported by the National Digital Agenda 2022–2026, which modernizes public services, including the healthcare system, in Albania. Over 95% of these services are currently offered online through the e-Albania portal [11].

On the other hand, Albanian nurses and nursing students recognize the crucial role of informatics in nursing. They highlighted their readiness to engage with technology in clinical practice [12]. According to the scientific literature, several research tools have been developed to explore eHealth literacy and perceived digital skills [13]. The Digital Health Literacy Instrument and the Electronic Health Literacy Assessment Toolkit are two that have been implemented in different populations, including undergraduate nursing students [14,15]. However, the eHealth Literacy Scale (eHEALS) remains the most widely accepted research tool for assessing eHealth literacy [13,16].

In 2019, Anderberg et al. developed an advanced research tool that incorporated the eHEALS [17]. Their multidimensional tool was designed to investigate nursing students’ eHealth literacy, their knowledge of Internet and technology device use, and their attitudes toward the necessity of eHealth literacy in their studies and future professions. It is a highly promising questionnaire as it explores a wide range of elements and core competencies among nursing students.

Internationally, four research teams have also used the initial tool designed by Anderberg et al. and have published their findings, providing a basis for cross-cultural comparison [18,19,20,21]. Lekalakala-Mokgele et al. (2023) conducted their survey, recruiting 244 undergraduate nursing students from the Department of Nursing in Gauteng province (South Africa) [18]. Their findings showed that nursing students’ eHealth literacy was assessed as moderate. Additionally, frequent use of technology in daily life was associated with greater confidence in using digital health tools [18]. Respectively, Andersson et al. (2023) utilized the same research tool to assess the knowledge and attitudes of 304 Polish and 342 Swedish nursing students toward technology and eHealth [19]. Participants’ eHealth literacy varied from moderate to high, with Swedish students scoring higher than the Polish students. The more students were engaged with technology, the more they recognized the positive impact of eHealth on their education and clinical practice. Moreover, Dallora et al. (2024) evaluated the perceptions and skills regarding technological devices, Internet use, and eHealth literacy by applying the same tool [20]. Their study also assessed nursing students’ anxiety and enthusiasm in engaging with technology, showing a positive association between greater confidence and active engagement [20]. A recent study among Albanian undergraduate and postgraduate health students, including 137 nursing students from the University of Shkodra “Luigj Gurakuqi”, examined their enthusiasm and anxiety toward technology, eHealth literacy, and digital skills [21]. Participants possessed adequate eHealth literacy, while their significant tech enthusiasm and notable digital skills were the main factors shaping and predicting eHealth literacy. However, their academic year of study did not impact their eHealth literacy, tech enthusiasm, tech anxiety, or skills in engaging with technology, such as Internet use frequency. Additionally, no statistically significant relationship was observed between male and female students [21].

Earlier in 2023, Shkurti and Shtiza investigated the behavior of Albanian health sciences students regarding the frequency and duration of Internet use, as well as the methods and reasons for accessing the Internet [22]. A total of 405 students were included in this survey, with the majority being nursing students (43.7%). The research showed that 28.2% of the participants spent 1–2 h per day surfing the net, especially for educational purposes, via Wi-Fi or mobile data in their leisure time. It is noteworthy that almost 19.3% reported browsing every moment. These studies underscore the growing international interest in eHealth literacy, pre-existing skills, and knowledge regarding technology engagement and the value of validated assessment tools such as the one designed by Anderberg et al. (2019) [17].

Regarding health literacy among Albanian undergraduate students, previous research has shown low overall literacy levels. Health science students (nursing, midwifery, physiotherapy, and laboratory technicians) from the Faculty of Medical Technical Sciences in Tirana reported that approximately 25% had inadequate or problematic health literacy [23]. Although health literacy and eHealth literacy are theoretically related, eHealth literacy requires both digital skills to access health-related information through electronic sources and the ability to assess the quality of that information [24,25]. Zerilli et al. (2026) conducted a cross-sectional study among 600 Italian nursing students and used the eHEALS and the Health Literacy Questionnaire to assess the students’ knowledge, attitudes, and skills regarding these topics [26]. Even though the findings of this study revealed that participants had adequate health literacy, they had lower eHealth literacy, underscoring the need to investigate both fields further, independently or in combination [26].

To our knowledge, no previously published validation studies or questionnaires suitable for implementation in the Albanian context exist, such as the one developed by Anderberg et al. (2019) [17]. Therefore, this study aimed to address this gap by translating, culturally adapting, and validating a questionnaire focused on technophilia, Internet usage, eHealth literacy, and nursing students’ attitudes toward technology and eHealth. This study seeks to provide preliminary evidence for a valid and reliable tool in the Albanian region. Assessing these parameters provides a foundation for nursing educators to tailor future strategies to enhance Albanian nursing students’ digital skills and eHealth literacy.

## 2. Materials and Methods

### 2.1. Design and Setting

This study was designed and conducted at the Faculty of Health of the University of Vlora “Ismail Qemali” in Albania (Vlora, Albania). The university offers a three-year Bachelor’s degree program in Nursing. A convenience sampling method was used to recruit the participants. Data for this cross-sectional validation study were collected from January to April 2022. The sample included undergraduate nursing students. Almost 1100 nursing students were invited to participate. Students received an online questionnaire via their institutional email addresses. Eligibility criteria were: (1) being an undergraduate nursing student enrolled in the three-year bachelor’s program at the Faculty of Health, University of Vlora “Ismail Qemali”; (2) having access to their official university email; and (3) providing signed informed consent. Students who were not actively attending courses or who had incomplete questionnaires were excluded. STROBE guidelines for cross-sectional studies were followed in this study [27]. A completed STROBE statement is provided in the Supplementary Materials.

### 2.2. The Questionnaire

The survey tool explores nursing students’ Internet use, including how often they surf the web, the devices they typically use, and the reasons for their web browsing. Along with the questionnaire, the evaluation assesses their knowledge and skills in using various forms of eHealth in their daily lives as nursing undergraduates and in their future professional roles. The tool comprises 30 items, organized into four subscales: Technophilia, Internet Use, Electronic Health Literacy Scale, and Technology and Electronic Health in Nursing Education [17].

In the first part of the questionnaire, participants completed sociodemographic questions regarding their age, gender, and semester of study. In the second part, the 30 items were separated into four subscales as follows: (a) Technophilia (nine questions), (b) Internet Use (seven questions), (c) Electronic Health Literacy Scale (eight questions), and (d) Technology and Electronic Health in Nursing Education (six questions). The Technophilia, Electronic Health Literacy Scale, and Technology and Electronic Health in Nursing Education subscales have a five-point Likert-type scale, where one corresponds to “Strongly Disagree” and 5 to “Strongly Agree”. The Internet Use subscale is assessed with a 6-point Likert scale, where one reflects “Several times daily” and six reflects “Never”, encouraging participants to make a more definitive choice regarding their frequency of Internet and technological device use (e.g., tablet, smartphone). A total score for each subscale was calculated.

The 30-item questionnaire used in this study was originally developed by Anderberg et al. (2019) [17] and consisted of four subscales. Particularly, to explore the levels of Technophilia, the TechPH questionnaire was used [28], while eHealth literacy was measured using the eHEALS scale [24]. Additionally, the Internet Use and Technology and Electronic Health in Nursing Education scales were also part of the original version of the tool [17].

### 2.3. Translation

Prior to the translation process, permission to use the instrument was obtained from the original authors. The translation of the questionnaire into the Albanian language was carried out in two directions (forward and backward translation) [29]. Initially, two independent bilingual translators translated the questionnaire from Swedish into Albanian (forward translation). One translator was an Albanian advanced registered nurse fluent in Swedish, and the other was a professional Albanian translator. Upon the completion of this phase, a meeting was organized between the two translators and an expert panel to reconcile and incorporate their versions into the Albanian version, enabling comparison and resulting in the first unified Albanian version of the questionnaire. The expert panel consisted of three clinical nurses, three professors of nursing, and an expert in psychometrics.

Next, a third professional translator, a native Swedish speaker fluent in Albanian, received the Albanian version. This translator had no prior involvement and was blinded to the original version. The expert produced an independent backward translation into Swedish. After that, the backward translation was compared with the original version by the expert panel. Then, they developed a second, revised version of the Albanian questionnaire.

Then, the Albanian translated version of the questionnaire was completed by 10 nursing students from the population of interest to assess its face validity, confirming that the scale consisted of consistent questions and did not elicit incomplete responses or misleading answers [30]. During the cultural adaptation process, the expert panel reviewed all instrument items for accuracy, clarity, and suitability in the Albanian context. Additionally, feedback from the participants regarding the clarity and comprehension of the items was sought. In this pre-testing phase, these comments were evaluated by the expert committee. No modifications were required, and the final Albanian version of the tool was produced.

### 2.4. Reliability-Validity

Cronbach’s alpha index was used to evaluate internal consistency. Reliability coefficients exceeding 0.70 indicate acceptable measurement stability and suggest that the items are internally consistent and conceptually cohesive, reflecting a single underlying construct [31].

EFA was conducted to investigate the instrument’s structure. The analysis was performed on the 30 items of the Albanian version using Equamax rotation to identify the underlying dimensions. To assess the suitability of the data for factor analysis, Bartlett’s test of sphericity was used to examine inter-item correlations. At the same time, the Kaiser–Meyer–Olkin (KMO) measure was used to evaluate sampling adequacy. A KMO value above 0.60 is considered acceptable for proceeding with factor analysis [32].

For factor retention, the final model was determined based on the following criteria: (i) scree plot analysis, retaining factors appearing before the inflection point (elbow) in the curve [33]; (ii) each factor must comprise at least three items with loadings ≥ 0.40 [34]; (iii) the total variance explained by the retained factors was also taken into account with a desired level of 75% or higher [35], although in social and behavioral sciences, explained variance levels around 50–60% may be considered as acceptable depending on the theoretical interpretability [36]; and (iv) the Kaiser criterion was used with eigenvalues to be greater than 1 [37].

Confirmatory factor analysis (CFA) was subsequently performed to assess the factor structure identified through EFA. The satisfactory or great fit of hypothesized relationships among constructs was shown by a standardized root mean square residual (SRMR) less than or equal to 0.08 [38], a comparative fit index (CFI) [38], and a Tucker–Lewis index (TLI) greater than or equal to 0.95 [39].

### 2.5. Statistical Analysis

All statistical analyses were conducted via statistical software IBM© SPSS© version 29 (IBM Corp. Released 2023. IBM SPSS Statistics for Windows, Version 29.0.2.0 IBM Corp, Armonk, NY, USA) and R Statistics software version 4.0.3 [40] using the lavaan package [41] and lavaanPlot package [42]. The statistical significance level was set at 0.05. The sample size was considered adequate based on commonly recommended item-to-participant ratios for factor analysis (typically 5–10 participants per item), which were satisfied in the present study [30]. EFA was assessed using the Equamax rotation method. The number of retained factors was determined using the Kaiser criterion (eigenvalues > 1), a scree plot, and interpretability of the factor solution. Internal consistency was evaluated using Cronbach’s alpha coefficient [43]. CFA was conducted within the SEM framework. Model fit was assessed using the comparative fit index (CFI), Tucker–Lewis index (TLI), and standardized root mean square residual (SRMR).

### 2.6. Ethical Considerations

Permission to use the questionnaire was obtained by email from the original author of the instrument. The University of Vlora “Ismail Qemali” Ethics Committee reviewed and approved this research with number 148/1/26-07-2021. This survey was conducted in full compliance with the new General Data Protection Regulation (GDPR) [EU 2016/679] as of 25 May 2018 regarding sensitive personal data. All participants were informed, through an informed consent procedure, that their answers would be used only for research purposes and that anonymity would be guaranteed in the final data reports.

## 3. Results

A total of 357 undergraduate students participated in this validation study. Students who were not actively attending courses during the data collection period (January–April 2022) and those who did not complete the questionnaire or provided incomplete responses were excluded. The demographic characteristics of the sample are presented in Table 1. The sample consisted of 295 females (82.6%) and 60 males (16.8%), with 2 participants (0.6%) not identifying their gender. The mean and standard deviation (SD) of age was 22.25 ± 7.36 years, with a range of 18 to 53 years. Most of the sample consisted of students in their first to third semesters (190 students, 53.2%), while 167 participants (46.8%) were in their fourth to ninth semesters.

The translated version of the tool was first pilot-tested on a small, representative sample of 10 nursing students from the target population. Participants reported that the items were easy to understand and culturally appropriate. No ambiguity or misunderstanding was noted, and no items were considered problematic. Since no modifications were needed, the translation was considered to have preserved the conceptual integrity of the initial scale.

Construct validity was assessed using EFA. All items had acceptable measures of sampling adequacy (MSA) values over 0.5, except for the last item [D6—“I think there are many other fields that are more important for a nurse” (0.414)] of the fourth subscale, Technology and Electronic Health in Nursing Education, which was excluded prior to the EFA. Item D6 was removed due to its low measure of sampling adequacy (MSA), indicating poor sampling adequacy and weak integration within the factor structure. In addition to statistical criteria, the item demonstrated limited conceptual alignment with the emerging factors, supporting its exclusion.

**Figure 1:**
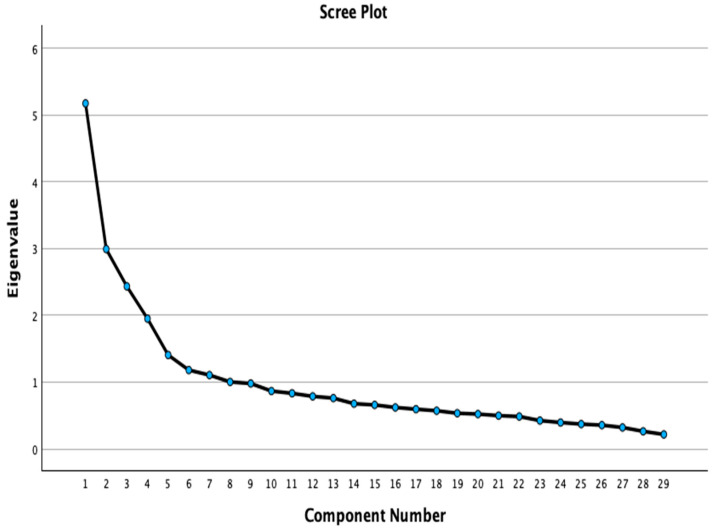
Scree plot of exploratory factor analysis.

A new EFA was conducted, which yielded a KMO value of 0.784 and a Bartlett’s Test of Sphericity of 2325.69 (*p* < 0.001). Four components were extracted after Equamax rotation; all had eigenvalues over 1 (4.09, 3.02, 2.93 and 2.51, respectively, for all components), and the cumulative explained variance was 53.29%, which represents a moderate but acceptable level in the context of behavioral and social science research when supported by additional criteria [36]. The scree plot (Figure 1), Kaiser criterion eigenvalues greater than 1 [37], and minimum explained variance (over 50%) indicated that the optimum number of factors was 4.

In Table 2, descriptive statistics for each item (mean, SD) are presented, as well as EFA indices (MSA, factor loadings) and reliability analyses (Cronbach’s alpha). Items A-2, A-4, and A-8 yielded negative responses to Technophilia, so reverse-scoring was required.

The internal consistency of the four scales was assessed using Cronbach’s alpha, which ranged from 0.692 to 0.852: Technophilia = 0.732; Internet Use = 0.695; Electronic Health Literacy Scale = 0.852; and Technology and Electronic Health in Nursing Education = 0.692, respectively.

**Figure 2:**
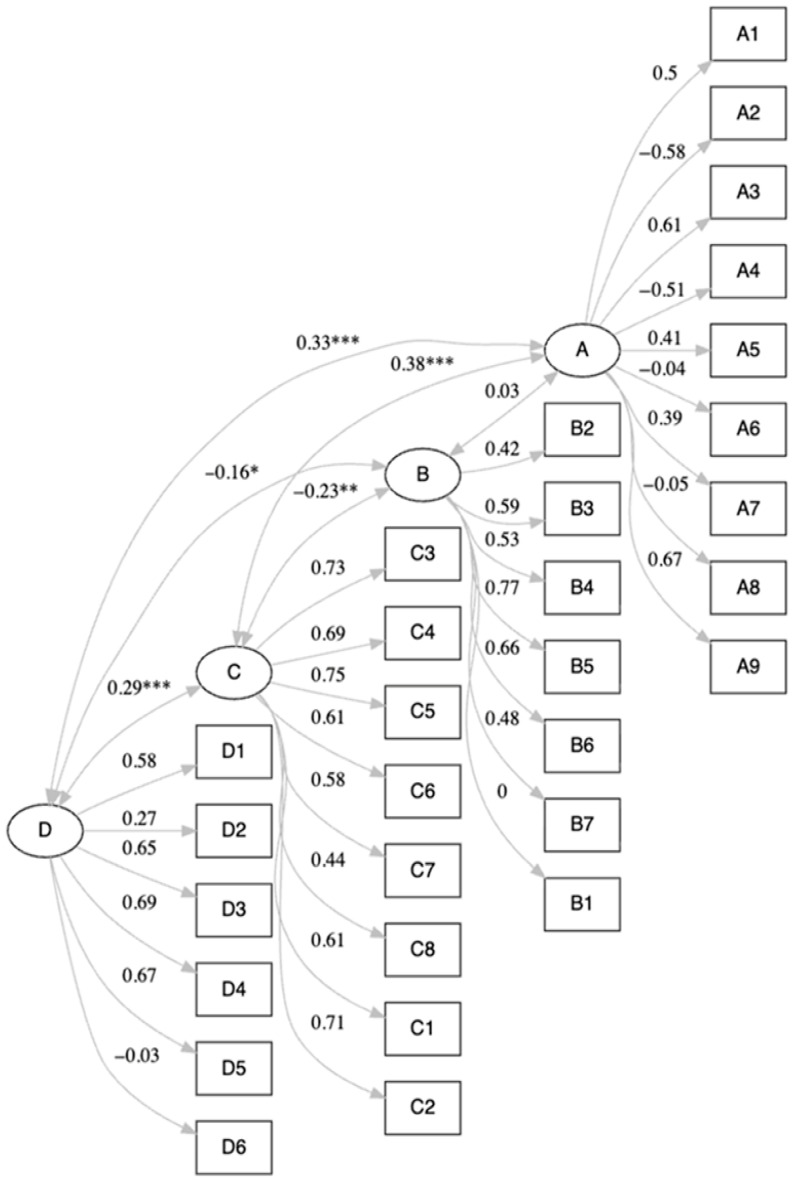
Structural equation modeling (SEM) of the Albanian version of the tool. Notes: * *p* < 0.05, ** *p* < 0.01, *** *p* < 0.001.

The four-factor model proposed by the developers of the instrument, along with the hypothesized relationships among the constructs, was examined using SEM (Figure 2). Fit indices indicated poor overall model fit (SRMR = 0.080, CFI = 0.772, TLI = 0.752). The findings from the SEM indicate that construct D exerted a strong and statistically significant impact on construct C (Electronic Health Literacy Scale; β = 0.29; *p* < 0.001) and that construct D (Technology and Electronic Health in Nursing Education) had a direct positive effect on construct A (Technophilia; β = 0.33, *p* < 0.001). In addition, construct C (Electronic Health Literacy Scale) had a statistically significant (negative) effect on construct B (Internet Use; β = −0.23; *p* < 0.01), and construct B (Internet Use) had a statistically significant (positive) effect on construct A (Technophilia; β = 0.42; *p* < 0.05). Taken together, these findings demonstrate that construct A (Technophilia) is impacted by both direct and indirect paths through the downstream influence of constructs C (Electronic Health Literacy Scale) and B (Internet Use).

## 4. Discussion

This study translated, culturally adapted, and validated a questionnaire assessing technophilia, Internet use, eHealth literacy, and nursing students’ attitudes toward technology and eHealth among Albanian undergraduate nursing students. A total of 357 students participated; most were female, with a mean age of 22.2 years. Approximately 53% were enrolled in their 1st–3rd semesters. The results showed generally satisfactory internal consistency for the instrument, with Cronbach’s alpha values ranging from 0.692 to 0.852 across the four subscales. Most subscales exceeded the commonly recommended threshold of 0.70. However, the lowest value (0.692) was slightly below this cutoff and should be interpreted with caution. These values were slightly lower than those reported by Lekalakala-Mokgele et al. (2023) [18]. That study reported the Cronbach’s alpha for the tool’s four scales, ranging from 0.71 to 0.90 [18]. In their recent study on Albanian nursing students, Fresku et al. (2026) reported Cronbach’s α values for three subscales (tech enthusiasm, tech anxiety, and eHEALs) of the tool ranging from 0.71 to 0.89 [21]. Such differences may relate to demographic, educational, and cultural variations between the populations. A comprehensive psychometric evaluation of the full instrument, including all four subscales and additional validity analyses, has not yet been fully reported. The present study addresses this gap by providing a more complete assessment of the instrument’s measurement properties.

In the Albanian version of the original 30-item tool, all items showed acceptable sampling adequacy (MSA > 0.5), except for one. The excluded item was D6: “I think there are many other areas that are more important for a nurse to gain more knowledge about than eHealth”. This item belongs to the Technology and Electronic Health in Nursing Education subscale. The exclusion may relate to differences in the curricula of Swedish and Albanian nursing departments. Specifically, the 3-year BSc Nursing program at the University of Vlora “Ismail Qemali” does not include lectures on nursing informatics or eHealth. In contrast, eHealth topics are part of the 3-year curricula at Blekinge Institute of Technology in Karlskrona and the Swedish Red Cross University College, from which the initial study’s sample was recruited, incorporating eHealth topics into their 3-year curricula, with mandatory eHealth lectures in the third semester and optional courses in the fifth semester [17]. Thus, Albanian nursing students, especially those in the early stages, may see eHealth as less important due to the limited emphasis in their education.

Although the overall model fit indices did not meet the commonly accepted cut-off values, the observed relationships were theoretically and conceptually meaningful. When the SEM model fit is poor on one or more fit indices, it is not necessarily considered theoretically invalid [44]. Therefore, the overall theoretical framework should also be considered when interpreting these results [44]. These findings suggest that the instrument may capture key dimensions of technophilia, eHealth literacy, and technology-related attitudes; however, they should be interpreted with caution. The SEM analysis indicated that technophilia (A) is influenced both directly by education (D) and indirectly through the sequential pathway D→C→B→A, indicating that the educational exposure first enhances nursing students’ competencies, subsequently shapes Internet usage patterns, and ultimately influences attitudes toward technology. The positive association between education and eHealth literacy (D→C; β = 0.29, *p* < 0.001) highlights the role of educational exposure in strengthening digital health competencies, consistent with prior work linking educational content to gains in eHealth literacy [45]. This correlation is clearly stated in the recent bibliography, where academic levels seem to be a predictor of eHealth literacy among nursing students [26]. The direct effect of education on technophilia (D→A; β = 0.33, *p* < 0.001) further indicates that educational experiences not only influence proficiencies, but also attitudes toward technology. This statement is supported by recent research demonstrating that nursing students’ technology proficiency and eHealth-related experiences are associated with more positive attitudes toward technology use. This also indicates that increased educational exposure and engagement with digital tools may foster a more favorable stance toward technology in nursing education [18,20,21]. Τhe negative association between eHealth literacy and Internet use (C→B; β = −0.23, *p* < 0.01) is in line with research showing that higher digital competencies are associated with more selective and purposeful Internet engagement patterns [46]. Finally, the strongest relationship in the model, between Internet use and technophilia (B→A; β = 0.42, *p* < 0.05), may reflect the importance of practical experience and user engagement in shaping positive attitudes toward technology, consistent with research showing that hands-on interaction supports the development of technological competence [20].

To date, the psychometric properties of this instrument in the Albanian context have not been formally reported. In a recent study, Fresku et al. (2026) used a modified version of the tool and reported Cronbach’s alpha values for selected subscales [21]. Given the absence of previously published exploratory or SEM analyses for earlier versions of the tool, direct psychometric comparisons across versions are limited. Nevertheless, the present study offers preliminary evidence regarding the validation of the Albanian version, a tool that may facilitate educators and researchers in assessing technophilia, eHealth literacy, and technology-related attitudes, addressing an important gap in nursing education research within the region.

This study had several limitations. First, the sample was drawn from a single university, limiting the generalizability of the findings to institutions nationwide. Second, discriminant validity and convergent validity were not formally assessed using other research tools, limiting the evaluation of the instrument’s construct validity. Third, the test-retest method was not employed to examine consistency over time as a measure of reliability. Fourth, the fit indices of the CFA indicated suboptimal SEM model fit, suggesting that the structural model should be interpreted cautiously. Additionally, content validity was evaluated based on the expert panel meeting outcomes rather than the commonly used content validity ratio (CVR) and content validity index (CVI).

Despite these limitations, the present study is the first attempt to examine the Albanian version of the tool using both EFA and SEM. Furthermore, this study provides a valuable contribution to efforts to explore technophilia, eHealth literacy, and technology-related attitudes in a previously understudied population. Future research should be based on the preliminary findings of this study to further evaluate the instrument’s psychometric properties in larger, more diverse samples. Then, tailored educational strategies should be developed, such as introducing digital health and eHealth lectures or courses, to accelerate the integration of eHealth and digital literacy into nursing curricula.

## 5. Conclusions

This study provides preliminary evidence on the reliability and validity of the Albanian version of the tool developed by Anderberg et al. (2019) [17]. Internal consistency of the instrument was generally satisfactory, with Cronbach’s alpha values ranging from 0.692 to 0.852 across the four subscales. Item-level analysis identified one item with lower sampling adequacy, suggesting curricular differences in eHealth education between Albanian and Swedish nursing programs. According to SEM analysis, technophilia is affected both directly and indirectly through eHealth literacy and Internet use, with educational exposure positively associated with eHealth literacy, and Internet use associated with higher eHealth literacy. These results should be interpreted cautiously due to suboptimal SEM fit and the lack of additional validity testing.

Overall, the findings indicate that the Albanian version is a preliminarily validated instrument that may serve as a useful tool to assess technophilia, eHealth literacy, and technology-related attitudes among Albanian nursing students. Further research should confirm its psychometric properties and its applicability across different institutions in Albania.

## Figures and Tables

**Table 1:** Respondents’ socio-demographic characteristics.

Variables		n (%)
Gender	Male	60 (16.8)
	Female	295 (82.6)
	Other	2 (0.6)
Age (22.25 ± 7.36 years)	18–19	142 (39.8)
	20–21	124 (34.7)
	22–23	42 (11.8)
	24+	49 (13.7)
Semester	1–3	190 (53.2)
	4–9	167 (46.8)

**Table 2:** Exploratory factor analysis for the Technology and Electronic Health Tool.

Item	Mean ± SD	MSA	Factor Loadings	Cronbach’s Alpha
Technophilia				0.732
A-1	3.81 ± 0.76	0.749	0.588	
A-2	2.09 ± 1.06	0.734	−0.567	
A-3	4.23 ± 0.73	0.827	0.666	
A-4	2.25 ± 1.11	0.775	−0.511	
A-5	3.28 ± 1.04	0.757	0.599	
A-6	2.98 ± 1.11	0.546	0.430	
A-7	3.44 ± 1.07	0.755	0.472	
A-8	3.64 ± 1.04	0.579	−0.410	
A-9	4.06 ± 0.91	0.815	0.597	
Internet Use				0.695
B-1	1.82 ± 1.16	0.714	0.473	
B-2	3.61 ± 1.71	0.699	0.551	
B-3	4.01 ± 1.47	0.835	0.639	
B-4	4.06 ± 1.80	0.831	0.573	
B-5	4.99 ± 1.25	0.717	0.767	
B-6	5.19 ± 1.32	0.759	0.716	
B-7	3.45 ± 1.45	0.816	0.548	
Electronic Health Literacy Scale				0.852
C-1	3.66 ± 0.77	0.886	0.647	
C-2	3.86 ± 0.69	0.808	0.698	
C-3	3.86 ± 0.69	0.768	0.752	
C-4	3.88 ± 0.74	0.878	0.695	
C-5	3.76 ± 0.73	0.863	0.756	
C-6	3.59 ± 0.83	0.800	0.642	
C-7	3.67 ± 0.81	0.817	0.628	
C-8	2.73 ± 1.08	0.857	0.538	
Technology and Electronic Health in Nursing Education				0.692
D-1	4.14 ± 0.70	0.784	0.603	
D-2	3.20 ± 0.87	0.774	0.380	
D-3	3.54 ± 0.78	0.718	0.719	
D-4	3.62 ± 0.87	0.651	0.775	
D-5	3.81 ± 0.84	0.734	0.717	

## Data Availability

The data presented in this study are available on request from the corresponding author.

## Supplementary Materials

The following supporting information can be downloaded at: https://www.mdpi.com/article/10.3390/nursrep16040139/s1, the completed STROBE checklist for this research study is provided as Supplementary Materials.

## Public Involvement Statement

No public involvement in any aspect of this research.

## Guidelines and Standards Statement

This manuscript was drafted using the “The Strengthening the Reporting of Observational Studies in Epidemiology (STROBE)” for reporting observational studies [27].
